# Association of dietary niacin intake with osteoporosis in the postmenopausal women in the US: NHANES 2007–2018

**DOI:** 10.3389/fmed.2025.1504892

**Published:** 2025-02-05

**Authors:** Li Li, Wankun Liang, Bing Deng, Yue Jiang, Xiaomin Huang, Yanlin Zhang, Tianrui Lu, Lu Wang, Yunxiang Xu, Guizhen Chen

**Affiliations:** ^1^The Seventh Clinical College of Guangzhou University of Chinese Medicine, Shenzhen Bao’an Traditional Chinese Medicine Hospital, Guangzhou University of Chinese Medicine, Shenzhen, China; ^2^Clinical Medical College of Acupuncture, Moxibustion and Rehabilitation, Guangzhou University of Chinese Medicine, Guangzhou, China

**Keywords:** niacin intake, osteoporosis, bone health, postmenopausal women, NHANES

## Abstract

**Background:**

Elderly individuals with inadequate vitamin B level are at increased risk of degenerative conditions, notably cardiovascular disorders, cognitive impairments, and osteoporosis. The relationship between niacin (vitamin B3) consumption and osteoporosis risk remains a subject of debate. This study aimed to clarify the relationship between dietary niacin intake and the incidence of osteoporosis in postmenopausal women aged ≥50 years.

**Methods:**

In this study, we gathered details on participants’ bone mineral density, osteoporosis status, dietary niacin intake, and several other critical variables. Multivariate logistic regression models were constructed to determine the association between dietary niacin intake and the incidence of osteoporosis. Restricted cubic splines were employed to further assess the linearity and explore the shape of the dose-response associations. Additionally, we performed stratified and interaction analyses to illustrate the stability of the observed relationships across different subgroups.

**Results:**

After adjusting for all covariates, there was a significant inverse association with osteoporosis (OR = 0.87; 95% CI: 0.77–0.97; *p* = 0.016). A negative relationship was observed between dietary niacin intake and the risk of osteoporosis (nonlinear: *p* = 0.672). While stratified analyses revealed some differences in the association between dietary niacin intake and osteoporosis risk, these differences were not statistically significant.

**Conclusion:**

Dietary niacin intake exhibited an inverse correlation with the incidence of osteoporosis. The risk of osteoporosis was significantly reduced by 13% with every 10 mg/day increase in daily dietary niacin consumption among postmenopausal women.

## 1 Introduction

Osteoporosis, a prevalent and debilitating skeletal disorder, is characterized by a reduction in bone mineral density, destruction of the bone microarchitecture, increased bone fragility and risk of fracture (1). Elderly individuals, particularly postmenopausal women, are at a significantly elevated risk for developing osteoporosis. Globally, the prevalence of osteoporosis among postmenopausal women is significantly high, reaching 27.4% (2). Fracture complications can significantly impair a patient’s ability to perform daily activities, which poses a substantial threat to the economy and healthcare infrastructure worldwide. Considering the severity and widespread nature of postmenopausal osteoporosis, identifying and effectively addressing the contributing risk factors is imperative for disease management.

Niacin, or vitamin B3, is an essential micronutrient predominantly derived from a variety of rich dietary sources, such as dairy, meat, seafood, legumes, cereals, and leafy vegetables. Moreover, humans are capable of synthesizing niacin *in vivo* through the tryptophan pathway (3, 4). Niacin serves as a dietary precursor essential for the synthesis of the coenzymes nicotinamide adenine dinucleotide (NAD) and nicotinamide adenine dinucleotide phosphate (NADPH), which are pivotal for numerous cellular redox reactions (3). Therefore, niacin may exert its potential osteoprotective effects through mitigating inflammation and modulating the expression of the NAD-dependent deacetylase sirtuin 1 (SIRT1) (5, 6).

In recent years, an increasing number of studies (7–9) have linked osteoporosis to nutrients (including niacin). However, the relationship between dietary niacin and osteoporosis risk in postmenopausal women still remains unclear. In this study, our hypothesis was that individuals with osteoporosis have lower dietary niacin intake, based on nutritional patterns observed in this population and findings from preceding related literatures. And we aimed to assess whether dietary niacin is associated with the incidence of osteoporosis in a relatively large and nationally representative population aged 50–80 years in the USA.

## 2 Materials and methods

### 2.1 Study population

**Figure 1:**
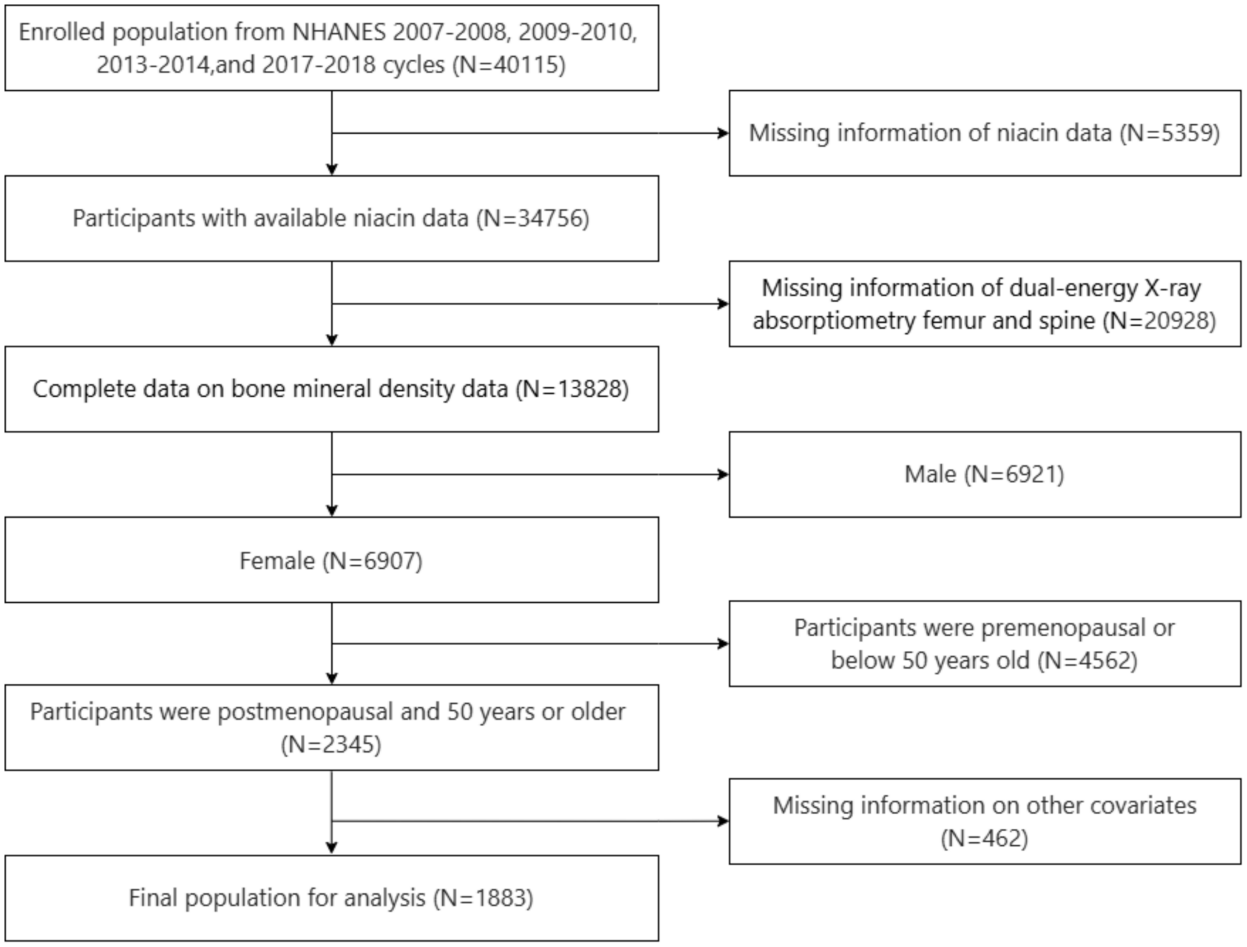
Flowchart of participant selection.

All subject information utilized in this cross-sectional study was extracted from the National Health and Nutrition Examination Survey (NHANES) conducted during the years 2007–2010, 2013–2014, and 2017–2018. The NHANES is a national continuous cross-sectional survey initiated by the National Centre for Health Statistics (NCHS), which aims to assess the health and nutrition of the U.S. civilian population. It collects comprehensive data through a stratified multistage probability sampling approach, with updates every two years (10). The NHANES provides valuable data for secondary analysis, contributing to insights into American Health and Nutrition. NHANES data are available on the NHANES website (http://www.cdc.gov/nchs/nhanes.htm; accessed on June 28, 2024). The NCHS Research Ethics Review Board approved the NHANES study protocol, and written informed consent was obtained from all participants before engaging in the study. The inclusion criteria were: population from NHANES 2007–2008, 2009–2010, 2013–2014, and 2017–2018; participants with available niacin data; participants with complete bone mineral density (BMD) data; postmenopausal women who were ≥50 years old. The exclusion criteria were: participants with missing dual-energy X-ray absorptiometry (DXA) data for the femur and spine; participants with missing data on other covariates. After the inclusion and exclusion criteria were applied, the final study cohort consisted of 1,883 participants (Figure 1).

### 2.2 Menopausal status definition

Menopausal status was ascertained through a self-administered reproductive health questionnaire in the NHANES survey. Women were classified as postmenopausal if they responded “no” to the question “have you had at least one menstrual period in the past 12 months? (Please do not include bleeding caused by medical conditions, hormone therapy, or surgeries.),” and selected either “hysterectomy” or “menopause/change of life” as the reason for amenorrhea. Further detailed information regarding the questionnaire is accessible in the NHANES database, which can be accessed through their official website (11).

### 2.3 Dietary niacin intake

The NHANES collects dietary intake data via the Computer-Assisted Dietary Interview (CADI) system, which is administered by bilingual (Spanish and English), trained interviewers. This comprehensive, computerized system employs a multiple-pass recall method to record the types and amounts of food and beverages consumed within a 24-h period prior to the interview. Each MEC dietary interview room is equipped with a standard set of measurement guidelines, developed with expert consensus to ensure accurate reporting of food volume and size. The NHANES Dietary Interviewer Procedure Manual provides a complete overview of these dietary survey methods (12). Niacin intake in this study refers exclusively to dietary sources and does not include supplemental niacin.

### 2.4 Determination of BMD and the diagnosis of osteoporosis

In the NHANES, BMD was assessed across various regions, including the total femur, femur neck, and total spine, utilizing DXA scans with Hologic QDR-4500A fan-beam densitometers (Hologic, Inc., Bedford, Massachusetts). The left hip was typically scanned, with exceptions made for subjects with a history of fracture, pin, or replacement on the left side, necessitating examination of the right hip. Participants were excluded from the DXA examination if they were pregnant; had a history of radiographic contrast material, bilateral hip fractures or replacements, or pins; or weighed over 450 lbs. Osteoporosis was diagnosed in accordance with the classification criteria established by the World Health Organization. When the BMD T-score in any region of the femur is ≤ −2.5, a value greater than 2.5 standard deviations below the reference mean is indicated for a young adult population (13, 14). Patients with osteopenia and those with normal BMD were considered as non-osteoporosis. Therefore, participants were also regarded as having osteoporosis if they answered “yes” to the question “Has a doctor ever told you that you had osteoporosis, sometimes called thin or brittle bones?” In cases where there is a discrepancy between the patient’s self-reported information and the data obtained from DXA scans, the diagnosis of osteoporosis should be based on the DXA scan data.

### 2.5 Covariates

On the basis of the NHANES website, we gathered covariate data through questionnaires, physical exams and laboratory testing. In this study, the following covariates were taken into account: age (year), race/ethnicity, education level (year), marital status, family poverty income ratio (PIR), smoking status, alcohol consumption status, body mass index (BMI) (kg/m^2^), physical activity, hypertension, diabetes, coronary heart disease (CHD), history of previous fractures, history of prednisone or cortisone, dietary supplements taken, blood calcium (mg/dL), and serum 25(OH)D (nmol/L) (15, 16). Race/ethnicity was classified as non-Hispanic White, non-Hispanic Black, Mexican American, or others. Education level was divided into three groups: less than 9 years, 9–12 years, and over 12 years. Marital status was categorized as living alone, married or living with a partner. Household income was stratified into low (PIR ≤1.3), middle (PIR 1.3–3.5), and high (PIR >3.5). Smoking status was defined as never (fewer than 100 cigarettes), current, or former (quit smoking after smoking 100+ cigarettes). Drinkers were defined as individuals who consume at least 12 alcoholic beverages per year. BMI was calculated using a standardized method based on weight and height measurements. Physical activity was labeled as sedentary, moderate (≥10 min of movement in the past 30 days causing only mild sweating or a slight to moderate increase in breathing or heart rate), or vigorous (≥10 min of activity in the past 30 days causing heavy sweating or a significant increase in breathing or heart rate). Previous diseases like hypertension, diabetes, and CHD were determined by questionnaire responses regarding physician diagnoses. The history of previous fractures, the use of prednisone or cortisone, and the intake of dietary supplements were classified as “yes” or “no” based on the participants’ reports. Blood calcium and serum 25(OH)D concentrations were determined via laboratory analysis of participants’ blood samples (12, 17).

### 2.6 Statistical analysis

Continuous variables with a normal distribution were presented as mean ± SD and contrasted through a one-way analysis of variance. Categorical variables were expressed as absolute values (percentages) and contrasted using Chi-square test. Multivariate logistic regression analysis was used to analyse the association between dietary niacin intake and osteoporosis while controlling for other covariates. The odds ratio (OR) and 95% confidence interval (CI) were used to reflect the direct relationship between dietary niacin intake and osteoporosis. Model 1 was adjusted for sociodemographic variables (age, marital status, race/ethnicity, education level, and PIR). Model 2 was further adjusted for smoking status, alcohol consumption status, BMI, physical activity, hypertension, diabetes, CHD, history of previous fractures, history of prednisone or cortisone, and dietary supplements taken. Model 3 was fully adjusted, similar to model 2, and additional adjustments for blood calcium and serum 25(OH)D. The restricted cubic spline (RCS) model was used to further assess the linearity and explore the shape of the dose-response relationship between dietary niacin intake and osteoporosis incidence. The smooth curve fitting graph was established and adjusted on the basis of the covariables contained in model 3. Moreover, logistic regression models were used to conduct interaction and subgroup analyses based on all covariates.

Given that the sample size was determined solely on the basis of the data provided, no *a priori* estimate of statistical power was made. All analyses were performed using the statistical software packages R 4.3.3 and Free Statistics software version 1.9.2 (18). A descriptive study was conducted on all the participants. A *p*-value of <0.05 indicated significance by a two-tailed testing.

## 3 Results

### 3.1 Baseline characteristics of the study population

A total of 1,883 qualified participants were included in this study. The prevalence of osteoporosis was 27.8% (523/1,883). Table 1 presents the characteristics of all the subjects with and without osteoporosis. The average age of the study population was 63.9 ± 8.6 years. Compared with participants without osteoporosis, osteoporosis patients often tended to be thinner and older, with a low or medium family income, coronary heart disease, a sedentary lifestyle, previous fractures, a history of prednisone or cortisone, higher consumption of dietary supplements, higher serum 25(OH)D level, and more likely to be non-Hispanic white. Notably, the non-osteoporosis population had a greater amount of dietary niacin.

**Table 1:** Baseline characteristics of the study participants.

Characteristics	All participants (*N* = 1,883)	Osteoporosis (*N* = 523)	No osteoporosis (*N* = 1,360)	*p*-value
Age (year), mean (SD)	63.9 (8.6)	67.4 (8.7)	62.5 (8.2)	<0.001
Race/ethnicity, *n* (%)				<0.001
Non-Hispanic White	899 (47.7)	302 (57.7)	597 (43.9)	
Non-Hispanic Black	355 (18.9)	59 (11.3)	296 (21.8)	
Mexican American	280 (14.9)	58 (11.1)	222 (16.3)	
Others	349 (18.5)	104 (19.9)	245 (18)	
Education level (year), *n* (%)				0.050
<9	202 (10.7)	56 (10.7)	146 (10.7)	
9–12	705 (37.4)	218 (41.7)	487 (35.8)	
>12	976 (51.8)	249 (47.6)	727 (53.5)	
Marital status, *n* (%)				0.081
Married or living with a partner	1,015 (53.9)	265 (50.7)	750 (55.1)	
Living alone	868 (46.1)	258 (49.3)	610 (44.9)	
PIR, *n* (%)				<0.001
Low	519 (27.6)	162 (31.0)	357 (26.2)	
Medium	737 (39.1)	227 (43.4)	510 (37.5)	
High	627 (33.3)	134 (25.6)	493 (36.2)	
Smoking, *n* (%)				0.198
Never	1,177 (62.5)	312 (59.7)	865 (63.6)	
Current	448 (23.8)	129 (24.7)	319 (23.5)	
Former	258 (13.7)	82 (15.7)	176 (12.9)	
Alcohol, *n* (%)				0.296
No	717 (38.1)	209 (40.0)	508 (37.4)	
Yes	1,166 (61.9)	314 (60.0)	852 (62.6)	
BMI (kg/m^2^), mean (SD)	28.5 (5.9)	26.5 (5.6)	29.2 (5.8)	<0.001
Physical activity, *n* (%)				0.021
Sedentary	1,113 (59.1)	328 (62.7)	785 (57.7)	
Moderate	574 (30.5)	156 (29.8)	418 (30.7)	
Vigorous	196 (10.4)	39 (7.5)	157 (11.5)	
Hypertension, *n* (%)				0.785
No	1,050 (55.8)	289 (55.3)	761 (56.0)	
Yes	833 (44.2)	234 (44.7)	599 (44.0)	
Diabetes, *n* (%)				0.300
No	1,553 (82.5)	439 (83.9)	1,114 (81.9)	
Yes	330 (17.5)	84 (16.1)	246 (18.1)	
CHD, *n* (%)				<0.001
No	1,805 (95.9)	486 (92.9)	1,319 (97)	
Yes	78 (4.1)	37 (7.1)	41 (3)	
History of previous fractures, *n* (%)				<0.001
No	1,387 (73.7)	356 (68.1)	1,031 (75.8)	
Yes	496 (26.3)	167 (31.9)	329 (24.2)	
History of prednisone or cortisone, *n* (%)				<0.001
No	1,731 (91.9)	462 (88.3)	1,269 (93.3)	
Yes	152 (8.1)	61 (11.7)	91 (6.7)	
Dietary supplements taken, *n* (%)				0.022
No	604 (32.1)	147 (28.1)	457 (33.6)	
Yes	1,279 (67.9)	376 (71.9)	903 (66.4)	
Blood calcium (mg/dL), mean (SD)	9.5 (0.4)	9.4 (0.4)	9.5 (0.4)	0.045
Serum 25(OH)D (nmol/L), mean (SD)	72.8 (30.9)	76.4 (30.3)	71.4 (31.0)	0.002
Niacin intake (mg/day), mean (SD)	19.4 (10.1)	18.3 (10.5)	19.9 (9.9)	0.003
TFBMD (gm/cm^2^), mean (SD)	0.8 (0.1)	0.7 (0.1)	0.9 (0.1)	<0.001
FNBMD (gm/cm^2^), mean (SD)	0.7 (0.1)	0.6 (0.1)	0.8 (0.1)	<0.001
TSBMD (gm/cm^2^), mean (SD)	0.9 (0.2)	0.8 (0.1)	1.0 (0.1)	<0.001

TF, total femur; FN, femoral neck; TS, total spine.

### 3.2 Relationship between dietary niacin intake and osteoporosis

**Figure 2:**
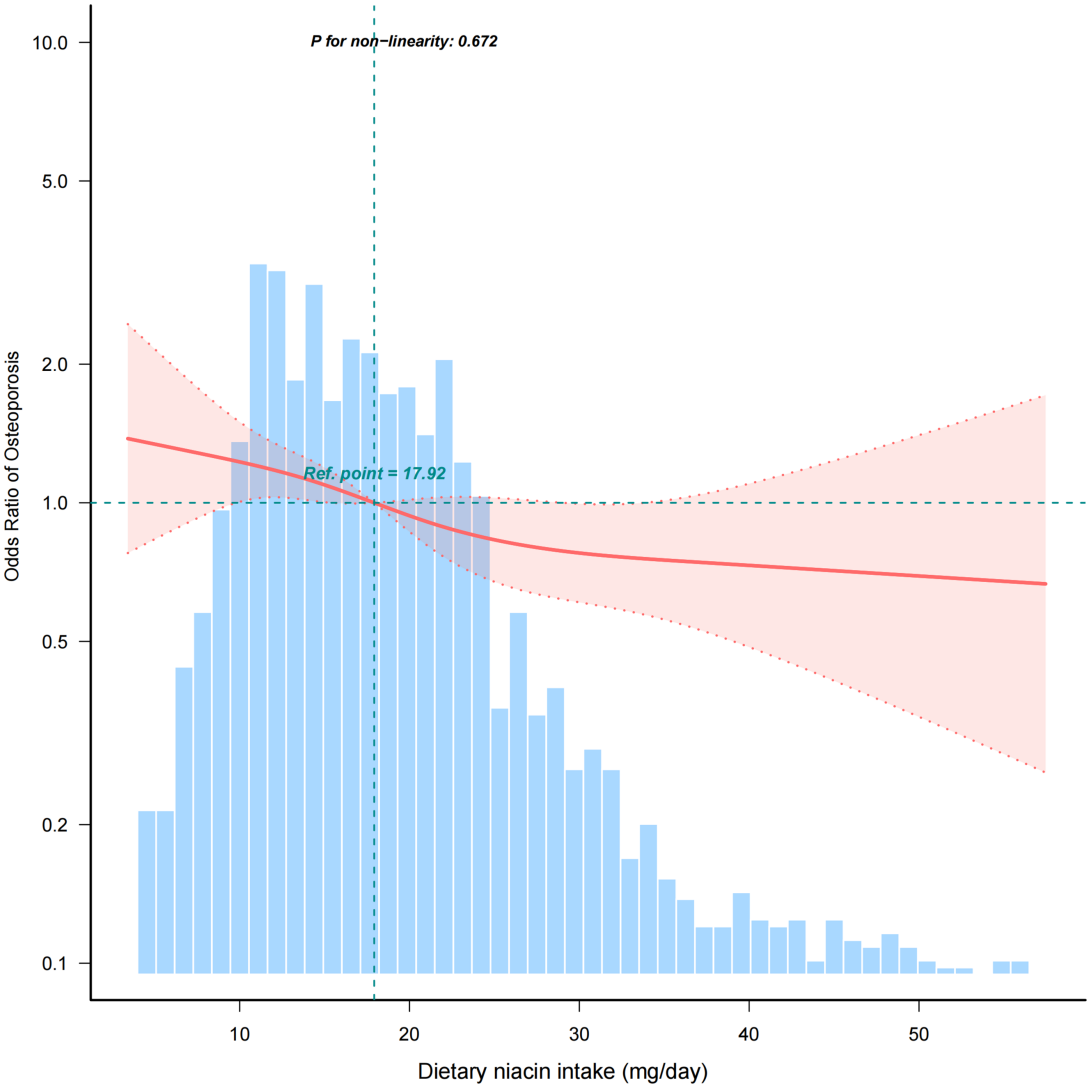
Association between dietary niacin intake and the osteoporosis odds ratio. The solid and dashed lines represent the predicted value and 95% confidence intervals, respectively. They were adjusted for age, marital status, race/ethnicity, education level, PIR, smoking, alcohol, BMI, physical activity, hypertension, diabetes, CHD, history of previous fractures, history of prednisone or cortisone, dietary supplements taken, blood calcium, and serum 25(OH)D. Only 99% of the data is shown.

Univariate regression analysis revealed that age, race and ethnicity, PIR, BMI, physical activity, CHD, history of previous fractures, history of prednisone or cortisone, dietary supplements taken, blood calcium, serum 25(OH)D, dietary niacin intake were significantly associated to osteoporosis (*p* < 0.05) (Supplementary Table). Besides, the relationship between dietary niacin intake and the risk of osteoporosis was also analysed by conducting multivariate logistic regression (Table 2). When niacin was analysed as a continuous variable, a significant independent negative association was discovered between dietary niacin intake and the risk of osteoporosis in the non-adjusted crude model (OR: 0.85, 95% CI: 0.76–0.95; *p* = 0.003); moreover, further adjustment did not significantly affect the results. Consistent findings were also obtained when niacin was analysed as a categorical variable. Compared with individuals with lower niacin consumption Q1 (<12.3 mg/day), the fully adjusted OR values for dietary niacin intake and osteoporosis in Q2 (12.4–18.3 mg/day), Q3 (18.4–26.2 mg/day), and Q4 (≥26.3 mg/day) were 0.82 (95% CI: 0.61–1.10, *p* = 0.191), 0.72 (95% CI: 0.53–0.97, *p* = 0.030), and 0.61 (95% CI: 0.43–0.85, *p* = 0.004), respectively. Accordingly, the RCS of the relationship between dietary niacin intake and osteoporosis was negative when all potential confounders were taken into account (nonlinearity: *p* = 0.672) (Figure 2).

**Table 2:** Association between dietary niacin intake and osteoporosis.

Variable	OR (95% CI)				
	No.	Crude	Model 1	Model 2	Model 3
Dietary niacin intake (per 10 mg/day)	1,883	0.85 (0.76–0.95)^**^	0.87 (0.77–0.97)^*^	0.87 (0.77–0.98)^*^	0.87 (0.77–0.97)^*^
Four group (mg/day)					
Q1 (≤12.3)	482	1 (Ref)	1 (Ref)	1 (Ref)	1 (Ref)
Q2 (12.4–18.3)	509	0.89 (0.68–1.17)	0.84 (0.63–1.12)	0.83 (0.62–1.11)	0.82 (0.61–1.10)
Q3 (18.4–26.2)	519	0.74 (0.56–0.98)^*^	0.72 (0.54–0.96)^*^	0.73 (0.54–0.98)^*^	0.72 (0.53–0.97)^*^
Q4 (≥26.3)	373	0.59 (0.44–0.81)^**^	0.6 (0.43–0.83)^**^	0.61 (0.44–0.86)^**^	0.61 (0.43–0.85)^**^
Trend test	1,883	<0.001	0.001	0.003	0.002

Crude model adjusted for: none. Model 1 adjusted for: sociodemographic variables (age, marital status, race/ethnicity, education level, PIR). Model 2 adjusted for: model 1 + smoking, alcohol, BMI, physical activity, hypertension, diabetes, CHD, history of previous fractures, history of prednisone or cortisone, and dietary supplements taken. Model 3 adjusted for: model 1 + model 2 + blood calcium, and serum 25(OH)D. Q, quartiles; OR, odds ratio; CI, confidence interval; Ref, reference. ^*^p < 0.05 and ^**^p < 0.01.

### 3.3 Subgroup analyses

**Figure 3:**
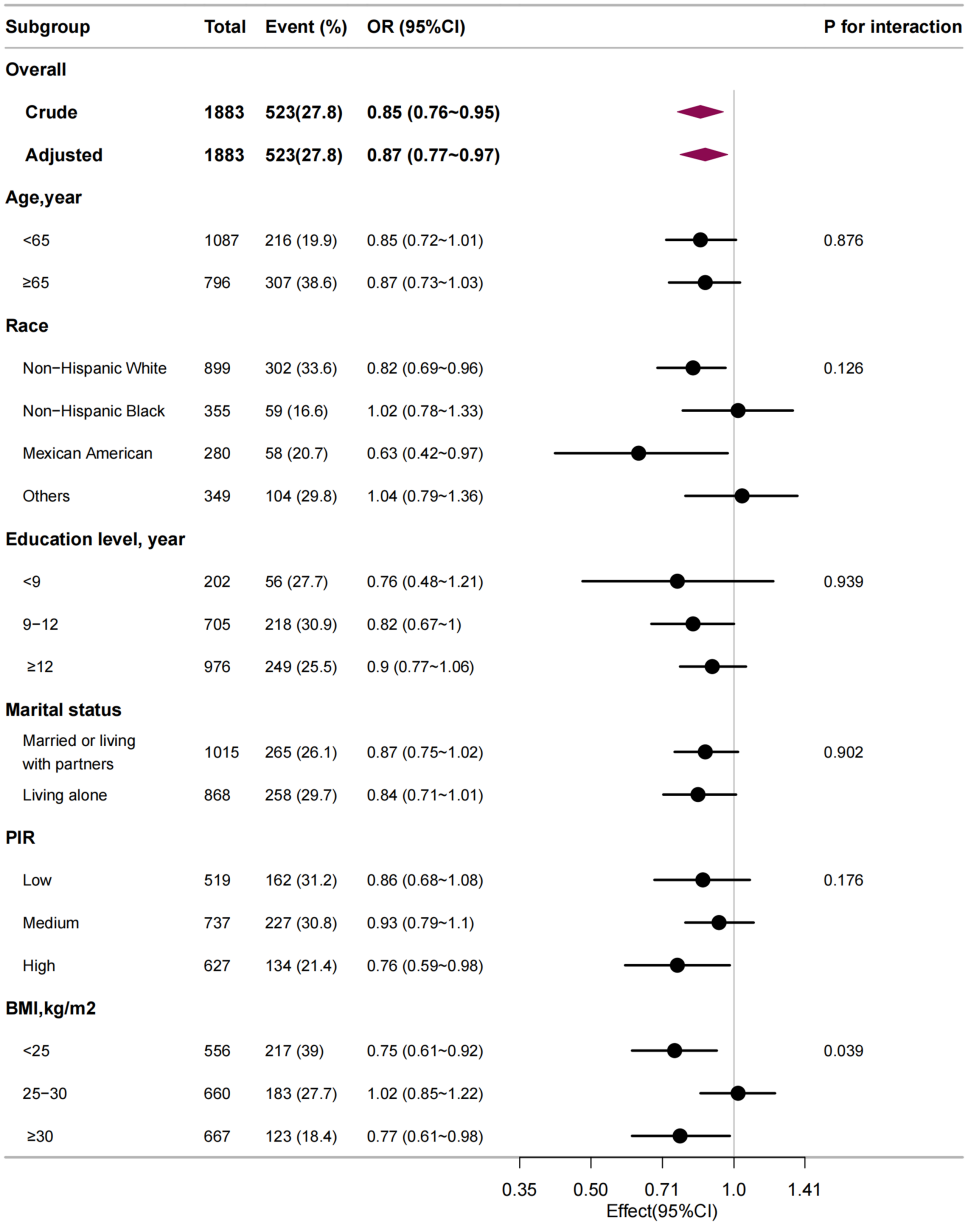
The relationship between dietary niacin intake (per 10 mg/day) and osteoporosis according to basic features. Each stratification factor was adjusted for all other variables (age, marital status, race/ethnicity, education level, PIR, smoking status, alcohol, BMI, physical activity, hypertension, diabetes, CHD, history of previous fractures, history of prednisone or cortisone, dietary supplements taken, blood calcium, and serum 25(OH)D).

Stratified and interaction analysis was performed to assess whether the association between dietary niacin intake and osteoporosis was consistent across several subgroups. However, there was no statistically significant interactions in any subgroups after stratifying by age, race/ethnicity, education level, marital status, PIR, or BMI (Figure 3).

## 4 Discussion

This nationally cross-sectional study of 1,883 American postmenopausal women from the NHANES (2007–2008, 2009–2010, 2013–2014, and 2017–2018) evaluated the association between dietary niacin intake and the incidence of osteoporosis. To our knowledge, this is the first study to explore the potential relationship between dietary niacin intake and osteoporosis risk in American postmenopausal women. After adjustment for potential confounders, dietary niacin was negatively associated with the incidence of osteoporosis. Moreover, when niacin was converted from a continuous variable to a categorical variable, the higher niacin quartile group (third quartile and fourth quartile) presented a lower osteoporosis risk than did the lowest niacin quartile group. Subgroup analysis further confirmed the stability of this association. Our findings highlight the importance of maintaining a niacin-rich diet in the prevention of osteoporosis among postmenopausal women.

Oxidative stress (OS) and inflammation are crucial pathogenic mechanisms underlying postmenopausal osteoporosis. Extensive animal researches have yielded robust evidence implicating reactive oxygen species (ROS) in the etiology of age-associated bone loss. Researchers have reported that ovariectomy-induced osteoporosis and osteoclast activity were markedly attenuated in mice deficient in key coenzymes necessary for ROS generation, such as nicotinamide adenine dinucleotide phosphate oxidase, or in those lacking antioxidants such as glutathione (19–21). After menopause, the body experiences an increase in the production of oxidizing substances, including ROS, malondialdehyde, and hydrogen peroxide (22–24). Concurrently, the activities of antioxidants, such as certain vitamins and folic acid, as well as those of antioxidant enzymes like superoxide dismutase, are significantly reduced (25). This leads to a disruption in the redox equilibrium of cells. The excessive accumulation of ROS can induce damage to lipids, proteins, and DNA in the cell membrane and nucleus. The OS state disrupts bone homeostasis by enhancing osteoclastogenesis, promoting apoptosis in osteoblasts and osteocytes, suppressing osteoblast activity, and inhibiting osteoprogenitor differentiation into the osteoblast cell lineage (26). OS enhances osteoclastogenesis through the upregulation of receptor activator of NF-kB ligand (RANKL) and the downregulation of osteoprotegerin (OPG), both of which are modulated via the Wnt/β-catenin pathway and facilitated by the activation of protein kinases (ERK1/2 and JNK) (27). SIRT1, a distinctive NAD^+^-dependent deacetylase, is among the most conserved members of the seven mammalian sirtuin family (28). It utilizes NAD^+^ to remove acetyl groups from proteins, particularly histone and non-histone acetylations, thereby regulating gene expression and protein function. As mentioned previously, niacin serves as a precursor of NAD and NADP and thus can elevate the level of NAD^+^ in the body, subsequently activating SIRT1. SIRT1 subsequently activates the Forkhead box O (FoxO)1/β-catenin signaling pathway through deacetylation, thereby mitigating oxidative stress-induced cellular damage from hydrogen peroxide (H_2_O_2_) and curbing apoptosis in osteoblasts (29, 30). This mechanism ultimately contributes to the therapeutic potential of SIRT1 in preventing osteoporosis.

In recent years, iron overload has been recognized as a new risk factor for postmenopausal osteoporosis (31, 32). Many researches have indicated that iron overload is prevalent among elderly individuals, particularly in postmenopausal women. Subsequent studies have shown that iron overload can lead to elevated levels of OS and ROS, which in turn compromises osteoblastogenesis and enhances osteoclastogenesis. This disruption in bone cell dynamics contributes to decreased bone mass and even osteoporosis (33, 34). Ma et al. (31) found that the primary cause of bone loss in Hepcidin^−/−^ mice was osteocyte apoptosis, which was attributed to elevated levels of ROS and subsequent alteration in sclerostin and RANKL/OPG expression. Moreover, the findings of Tao et al. (35) suggested that niacin, a potent antioxidant and anti-inflammatory agent, reduced oxidative stress and reversed bone loss caused by iron overload and estrogen deficiency through activating the SIRT1 signaling pathway. In their MC3T3-E1 cell experiment, niacin significantly reversed the adverse effects of iron overload on osteoblast activity and differentiation, as well as the protection of mitochondrial function and reduction in oxidative damage through the upregulation of SIRT1 and SOD2 expression. Additionally, niacin exerted a protective effect on bone health by inhibiting ferric citrate-induced osteoclast differentiation in RAW264.7 cells, further protecting bone health. From the preceding discourse, it is apparent that niacin may contribute to anti-osteoporosis by ameliorating oxidative stress, inflammation, and aging in the body, suggesting that dietary niacin could serve as a potential protective factor against osteoporosis. A modest-sized observational study (36) involving a cohort of 100 Polish women aged 51–70 years also revealed that osteoporosis patients had 16% lower niacin intake than non-osteoporosis individuals did. It is worth mentioning that although there was a negative linear relationship between dietary niacin intake and the risk of osteoporosis in this study, it should not be assumed that higher levels of dietary niacin supplementation are necessarily beneficial to human health. Although the essential requisites for NAD^+^ biosynthesis can be adequately met by consuming less than 20 mg of niacin daily, there is growing evidence indicating that significantly increasing the rate of NAD^+^ synthesis may be beneficial for certain degenerative diseases (37). Nevertheless, a nutritional review suggested that the upper limit of tolerable dietary niacin intake for adults is 35 mg/day (38). In addition, experimental studies in chicks have demonstrated that feeding high levels of supplemental niacin was found to be associated with adverse effects on bone strength, dimensions, and susceptibility to fracture (39, 40). And Table 1 indicates that the use of dietary supplements is positively correlated with the incidence of osteoporosis, suggesting the importance of taking dietary supplements in a reasonable and moderate manner. A meta-analysis suggested that the optimal intake levels may vary between healthy individuals and those with specific disease conditions. It prompted a re-evaluation of the dosage thresholds for nicotinic acid supplements (41). Nevertheless, our findings indicate that, within reasonable limits, increasing dietary niacin intake may exert a protective impact on the prevention of osteoporosis. Future prospective researches are warranted to confirm the potential preventative role of niacin in osteoporosis and its underlying mechanisms.

Our study has several noteworthy strengths. First, to our knowledge, this is the largest investigation to date exploring the relationship between dietary niacin intake and the risk of osteoporosis in postmenopausal women, thereby comprising participants from various ethnic backgrounds. Second, the adoption of a large-scale sample design from a nationally representative cohort of U.S. adults, ensured a robust population size. Third, by adjusting for potential confounding factors, including a history of prednisone and dietary supplements, our conclusions are more plausible and persuasive. Notwithstanding the advantages, there are still some limitations that require attention. First, the cross-sectional nature of our study prevents us from drawing conclusions regarding the causal relationship between dietary niacin intake and postmenopausal osteoporosis. Second, our study population may not be fully representative of all demographic groups, which could affect the generalizability of our findings. Third, despite our efforts to control for confounding factors, residual confounding cannot be entirely ruled out. Fourth, the dietary intake assessment in the NHANES, which relies on 24-h recall questionnaires, may be subject to inevitable recall bias. Nonetheless, the food frequency questionnaire, while a valuable tool, offers less comprehensive information regarding food types and quantities than does 24-h dietary recall (42, 43). Fifth, we have included data on dietary niacin intake only and have not incorporated data on supplemental niacin. Hence, it is imperative for future multicentre, large-sample cohort studies to validate these findings by incorporating more potential confounders and employing standardized measures.

In conclusion, dietary niacin intake was negatively associated with the risk of osteoporosis in postmenopausal women. Dietary niacin intake and the incidence of osteoporosis were negatively associated; the risk of osteoporosis was significantly reduced by 13% with every 10 mg/day increase in daily dietary niacin consumption.

## Data Availability

Publicly available datasets were analyzed in this study. This data can be found at: https://wwwn.cdc.gov/nchs/nhanes/Default.aspx.
