# Supplementary material for: Association of dietary niacin intake with osteoporosis in the postmenopausal women in the US: NHANES 2007–2018
Source: Front Med (Lausanne). 2025 Feb 5;12:1504892. doi: 10.3389/fmed.2025.1504892 (PMC11835798; doi:10.3389/fmed.2025.1504892)
Supplement: Supplementary file 1 [file Table_1.docx]

**Table1.** Univariate regression analysis.

| **Variable** | **OR (95% CI)** | ***p*-value** |
| --- | --- | --- |
| Age (year) | 1.07 (1.06-1.08) | < 0.001 |
| Race/ethnicity, n (%) |  |  |
| Non-Hispanic white | 1 (Ref) |  |
| Non-Hispanic black | 0.39 (0.29-0.54) | <0.001 |
| Mexican American | 0.52 (0.37-0.71) | <0.001 |
| Others | 0.84 (0.64-1.1) | 0.199 |
| Education level (year), n (%) |  |  |
| <9 | 1 (Ref) |  |
| 9-12 | 1.17 (0.82-1.65) | 0.383 |
| >12 | 0.89 (0.64-1.25) | 0.514 |
| Marital status, n (%) |  |  |
| Married or living with a partner | 1 (Ref) |  |
| Living alone | 1.2 (0.98-1.46) | 0.081 |
| PIR, n (%) |  |  |
| Low | 1 (Ref) |  |
| Medium | 0.98 (0.77-1.25) | 0.876 |
| High | 0.6 (0.46-0.78) | <0.001 |
| Smoking, n (%) |  |  |
| Never | 1 (Ref) |  |
| Current | 1.12 (0.88-1.43) | 0.354 |
| Former | 1.29 (0.96-1.73) | 0.086 |
| Alcohol, n (%) |  |  |
| No | 1 (Ref) |  |
| Yes | 0.9 (0.73-1.1) | 0.297 |
| BMI (kg/m2), Mean (SD) | 0.92 (0.9-0.94) | <0.001 |
| Physical activity, n (%) |  |  |
| Sedentary | 1 (Ref) |  |
| Moderate | 0.89 (0.71-1.12) | 0.324 |
| Vigorous | 0.59 (0.41-0.86) | 0.006 |
| Hypertension, n (%) |  |  |
| No | 1 (Ref) |  |
| Yes | 1.03 (0.84-1.26) | 0.785 |
| Diabetes, n (%) |  |  |
| No | 1 (Ref) |  |
| Yes | 0.87 (0.66-1.14) | 0.3 |
| CHD, n (%) |  |  |
| No | 1 (Ref) |  |
| Yes | 2.45 (1.55-3.87) | <0.001 |
| History of previous fractures, n (%) |  |  |
| No | 1 (Ref) |  |
| Yes | 1.47 (1.18-1.84) | 0.001 |
| History of prednisone or cortisone, n (%) |  |  |
| No | 1 (Ref) |  |
| Yes | 1.84 (1.31-2.59) | <0.001 |
| Dietary supplements taken, n (%) |  |  |
| No | 1 (Ref) |  |
| Yes | 1.29 (1.04-1.62) | 0.022 |
| Blood calcium (mg/dL) | 0.76 (0.58-0.99) | 0.046 |
| Serum 25(OH)D (nmol/L) | 1.01 (1-1.01) | 0.002 |
| Niacin intake (mg/d) | 0.98 (0.97-0.99) | 0.003 |

**Abbreviations:** OR, odds ratio; CI, confidence interval; PIR, family poverty income ratio; BMI, body mass index; CHD, coronary heart disease.
